# 

**Published:** 1893-07-01

**Authors:** 


**OUR NEW OFFICES**

428, Strand, W.C., will henceforth be the Office of this Journal.

**Notices of Births, Deaths, and Marriages,** are inserted in this column at a charge of 2s. 6d. for an announcement not exceeding four lines.

### NOTICE TO CORRESPONDENTS.

All MS., Letters, Books for Review, and other matters intended for the Editor should be addressed The Editor, The Lodge, Porchester Square, London, W.

The Editor cannot undertake to return rejected M.S., even when accompanied by stamped directed envelope.

**Notice to Advertisers and Subscribers.**

All Advertisements, Orders for Copies of The Hospital, and Business Communications should be addressed to The Publisher, *not to the Editor*, The Hospital, 428, Strand, W.C.

The Cost of Subscription, post free, is as follows:—Per week, 2½d.; per quarter, 2s. 6d.; per half-year, 5s.; per annum, 8s. 6d. Foreign:—Per annum, 10s. 8d.; payable, in all cases, in advance.

**"Burdett's Hospital Annual," 1893.**

Fourth year of publication. 700 pp. Boards, gilt lettering. A most complete guide to British, Colonial, Indian, and American Hospitals, Dispensaries, Nursing and Convalescent Institutions. and Asylums. Price 5s. Published by The Scientific Press (Limited), 428, Strand, W.C.

**NOTICE.—The Index for Vol. XIII. (ending March 1893) is on sale at the Office of this Journal, price 2½d., post free.**

### Scraps and Gleanings.

Dr. Kurth has been appointed director of the new Bacteriological Institute at Bremen.

During the year 1891 thirty persons died from starvation in London, none of them having applied for public relief.

A meeting of the women's branch of the Swanley Horticultural Society was held at 24, Park Lane on January 19th.

The North-Eastern Hospital for Children, Hackney Road, has received a donation of £200 from the trustees of Smith's Charity.

In a recent endemic of beri beri among Tonkinese convicts from the jail of Cochin China, out of 800 men 200 were afflicted and 70 died.

Every village in Algeria has to provide itself with a stock of locust ploughs in order to clear the roads of vast quantities of those troublesome insects.

The monster bazaar held in the beginning of last month in aid of the City of Dublin Hospital was a great success. The financial results surpassed the happiest expectations.

On Saturday, June 17th, the foundation-stone of the new infirmary at Halifax was laid by the Provincial Grand Master, after an especial Provincial Grand Lodge at the Masonic Hall.

A new game has been introduced into England from New Zealand called rounders on horseback. It is a game in which ladies can take part, and it has the charms of polo without the dangers.

There is a superstition among the inhabitants of the Caucasus that persons suffering from disease are possessed of angels, and that these are readily irritated, when it is a sad look-out for the patient.

The free library garden at Shoreditch is now completed and ready for use. A comfortable reading shelter has been erected for public use, and the garden is tastefully laid out with shrubs and flowers.

The annual meeting of the subscribers to the Hull, East Riding, and North Lincolnshire Orthopœdic Hospital was held on May 26th. The balance-sheet showed the expenditure over the receipts to be £20 2s. 7d.

The festival of the Miller Hospital and Royal Kent Dispensary is to be held on Tuesday, July 4th, at the Grand Hotel, Northumberland Avenue. On this and future occasions it is to take the form of a banquet.

At the monthly meeting in May of the Royal Hospital for Incurables at Dublin it was decided to erect another pavilion, to contain two wards, each for twenty-four beds. The cost, including furniture, will be about £6,000.

The new Convalescent Home for Nottingham and Notts was opened on the 3rd of last month by the Mayor of Nottingham. The building will accommodate thirty patients, and stands in its own grounds of three and a half acres.

Ivy House, York Road, Leeds, having been abandoned as a convalescent home for fever patients, was last month burned to the ground under the superintendence of the fire brigade in order to destroy all infectious germs lurking in the old house.

At a special meeting of the Limavady Guardians held on the 7th of last month a question arose regarding the erection of a cholera hospital. After some discussion it was decided that a building provided for twelve cholera patients should be arranged for.

The General Hospital at Wolverhampton is making an urgent appeal to the public for funds to the amount of £2,000 to meet the deficiency in the accounts, and the prospective outlay which is proposed in the building of a new mortuary and other necessary buildings.

Beside the ordinary cases of accident and emergency, which are always treated at the Seamen's Hospital, Royal Victoria and Albert Dock, E., special arrangements are being made to receive additional cases in July and August while the Poplar Hospital is closed.

An interesting feature at the Royal Horticultural Society in the Drill Hall, Westminster, consisted of blocks of ice from New Zealand, in which were embedded ferns and branches of flowering plants. On thawing, however, the flowers are stated to be quite destroyed.

Complaints are being made about the decision to exclude women from the clinical teaching in the Edinburgh Royal Infirmary. One contributor to the infirmary suggests that junior male students should be sent to the Leith Hospital, thus leaving more room for women at the Royal Infirmary.

The arrow poison used by the natives of the New Hebrides is made from earth from marshy places. It contains both the septic vibrio and the tetanus bacillus. From experiments on animals it was found that when the poison was old they died from tetanus, when fresh from septicæmia.

At a recent meeting of the Hull and Goole Port Sanitary Authority it was decided to purchase the hull of a vessel lying in the Thames for £500, with a view to using it as a floating cholera hospital, to be placed in the Humber, provided the Surveyor, on examination, reported the ship in a satisfactory condition.

A crab well known in English aquariums, and called the spider crab, is in the habit of concealing itself by decorating its spiny back with pieces of green seaweeds which it nips off with its claws. An observer at the Jersey Aquarium has seen this crab lift small stones on to its back until it lies concealed under a heap.

Her Royal Highness the Duchess of Teck attended a bazaar at the Royal Hospital for Incurables, West Hill, Putney Heath. Princess Victoria Mary of Teck was expected to be present, but was unable to attend. The Duchess of Teck made the tour of the bazaar, afterwards visiting several of the inmates.

Hospital Saturday at Birmingham was a great success. A special effort in the annual collection for the hospital realised the sum of £9,015. With the arrears of delayed contributions, this, it is expected, will bring up the total to £12,000. The strength of the collection lay in the penny-a-week system in the factories.

A collection amounting to £10 5s. has been made in small sums from Lowestoft fishermen for the Newlyn Nursing Fund. It was made in special recognition of the nurses' services to fishermen. At certain seasons of the year fishermen are liable to have their hands poisoned, and such and other misadventures have been greatly assuaged by the Newlyn nurses.

### BOOKS RECEIVED.

W. B. SAUNDERS, PHILADELPHIA.

"A Text Book of the Theory and Practice of Medicine." By William Pepper, M.D.

RELIGIOUS TRACT SOCIETY.

"Jessica's Mother." By Hesba Stretton.

OXFORD UNIVERSITY PRESS.

"Helps to the Study of the Bible."

GEORGE ROUTLEDGE AND SONS.

"The London Medical Student." By Albert Smith.

SIMPKIN, MARSHALL.
"The Disease of Inebriety." By the American Association for the Study and Care of Inebriety.

SWAN SONNENSCHEIN AND CO.

"Medical Electricity." By E. S. D'Odiardi.

THE VEGETARIAN PUBLISHING OFFICE.
"Sunshine and Shadow." By Arnold Frank Hills.

W. H. ALLEN AND CO. (LIMITED).

"Sir Morell Mackenzie: a Memoir." By the Rev. H. R. Haweis, M.A.

HODDER BROTHERS.
"Bright Thoughts for Weary Hours." By Clara Bray.

MACMILLAN AND CO.

"Lectures on Sanitary Law." By A. Wynter Blyth, M.R.C.S.

H. K. LEWIS.

"The Pathology of the Veriform Appendix." By T. V. Kelynack, M.D.

**Periodicals and Pamphlets.** — Food and Sanitation; The Medical Chronicle; The Whitehall Review; London; Toilers of the Deep; The Birmingham Medical Review; Reichs Medicinal Anzliger; The Strand Magazine; The Picture Magazine; The Clinical Journal; Talk; The Therapist; The Religious Review of Reviews.

The Religious Tract Society: The Leisure Hour, The Boy's Own Paper, The Girl's Own Paper and Summer Numbers, The Sunday at Home.

### Medical Vacancies and Appointments.

**APPOINTMENTS.**

J. Barclay, M.D., C.M.Aberd., reappointed Parochial Medical Officer of Health for Banff. J. H. Blayney, L.F.P.S.Glasg., reappointed Medical Officer of Health for the Prestwich Union Rural Sanitary Authority for Great and Little Heaton. E. G. Carter, M.R.C.S.Eng., appointed Medical Officer of the Industrial School, Moor Allerton, Leeds. F. B. H. Caudwell, L.R.C.P. Lond., M.R.C.S.Eng., appointed Medical Officer for the Coggeshall Sanitary District of the Braintree Union. J. Chadwick, M.R.C.S.Eng., L.S.A., appointed Medical Officer for the Butterworth Sanitary District of the Rochdale Union. J. J. Y. Dalgarno, M.B., C.M.Aberd., reappointed Medical Officer for the St. Nicholas Parish, Aberdeen. E. S. Dashwood, M.R.C.S.Eng., appointed Medical Officer for the Third District of the Aylsham Union. D. W. Davies, M.D., C.M.Aberd., reappointed Medical Officer for the Llanharran District of the Bridgend Union. G. H. Eccles, L.R.C.P.Edin., M.R.C.S., appointed Medical Officer to the Workhouse of the Braintree Union. G. S. Elliston, M.R.C.S.Eng., L.S.A., appointed Medical Inspector of Seamen for the Port of Ipswich. C. G. Gibbs, M.B., C.M.Edin., reappointed Medical Officer for the Launceston Urban Sanitary District. C. W. Glassington, M.R.C.S.Eng., L.D.S.Edin., appointed Dental Surgeon to the National Dental Hospital. J. Gordon, M.D., C.M.Aberd., reappointed Parochial Medical Officer for St. Nicholas, Aberdeen. J. Gordon-Smith, L.R.C.P., L.R.C.S.Edin., L.F.P. and S.Glasg., appointed Government Medical Officer for the Good Hope District, Trelawny, Jamaica, W.I. A. Hind, L.R.C.S., L.M.Edin., reappointed Medical Officer for the South Molton Urban Sanitary District. Dr. Holmes, appointed Medical Officer for the Penkridge District of the Cannock Union. S. Hughes, M.B., C.M., M.R.C.S., L.S.A., appointed on the Honorary Staff of St. Mark's Episcopal Hospital, Salt Lake City, Utah. J. Ironside, M.B., C.M.Aberd., reappointed Parochial Medical Officer for Garvoch. O. J. Kaufmann, M.D.Lond., appointed Professor of Pathology at Mason College, Birmingham. A. B. Lyon, M.B., C.M.Aberd., appointed pro tem. Medical Officer for the Macduff Sanitary District. N. S. Manning, L.R.C.P., F.R.C.S.I., appointed Medical Officer for the Combmartin Sanitary District of the Barnstaple Union. A. M. Palmer, L.R.C.P., L.M.Edin., M.R.C.S.Eng., appointed Medical Officer of Health for the Whittington Urban Sanitary District. A. A. Philip, M.B., C.M.Aberd., appointed Surgeon to the Naworth Collieries, Carlisle. D. Rennet, M.B., C.M.Aberd., reappointed Medical Officer to the City Parish, Aberdeen. H. W. Roberts, M.R.C.S.Eng., L.S.A., reappointed Medical Officer of Health for the Southwark Local Board. H. D. Rolleston, M.A., M.D.Camb., appointed Assistant Physician to St. George's Hospital. J. G. Scroggie, M.B., C.M.Aberd., reappointed Medical Officer for St. Nicholas Parish, Aberdeen. J. A. Shaw-Mackenzie, M.B.Lond., M.R.C.S., appointed Physician to the Out-patients to the Chelsea Hospital for Women. A. M. Sheild, M.B.Camb., F.R.C.S.Eng., appointed Assistant Surgeon to St. George's Hospital. F. M. T. Skae, M.B., C.M.Edin., appointed Junior Assistant Medical Officer to the Stirling District Asylum, Larbert. P. R. Stevens, L.R.C.P.Lond., M.R.C.S.Eng., appointed Medical Officer for the Bocking Sanitary District of the Braintree Union. S. T. Taylor, M.B., L.R.C.P.Lond., M.R.C.S. Eng., reappointed Medical Officer of Health for Cromer. W. Wyllie, M.D.Glasg., reappointed Medical Officer of Health for the Kirkby Lonsdale Urban Sanitary District. E. S. Yonge,

### MEDICAL VACANCIES AND APPOINTMENTS—(*continued*)

M.B., C.M.Edin., appointed House-Surgeon to the Manchester Southern and Maternity Hospital.

### VACANCIES.

The following vacancies are announced this week, the amount of salary in each case being given after the name of the institution:

**Resident Medical Officers.**

Central London Ophthalmic Hospital, 238A, Gray's Inn Road, N.W. House Surgeon. Rooms, coal, and light provided. Applications to the Secretary by July 10th.

East London Hospital for Children, Glamis Road, Shadwell, E. House-Surgeon. Board and lodging provided. Applications to the Secretary by July 6th.

Essex and Colchester General Hospital, Colchester. House-Surgeon; doubly qualified; unmarried or widower without a family. Salary £100 per annum, with board and lodging. Applications to the Committee before noon on July 8th.

General Hospital, Birmingham. Assistant House-Surgeon. No salary; residence, board, and washing. Applications, with registration certificate, to the House Governor by July 1st.

Jersey General Dispensary and Infirmary. Resident Medical Officer; unmarried; doubly qualified. Salary £120 per annum, with furnished lodgings, attendance, coal and gas. Applications to the Secretary, 20, Aquila Road, St. Heliers, Jersey, by July 6th.

Liverpool Dispensaries. Assistant Surgeon; unmarried. Salary £80 per annum, with apartments, board, and attendance. Applications to R. R. Greene, Secretary, by July 24th.

Monkwearmouth and Southwick Hospital. 5, Bedford Terrace, Sunderland. House-Surgeon; doubly qualified. Salary £80 per annum, with board, lodging, &c. Applications to Scott Gunn, Honorary Secretary, by June 30th.

Tunbridge Wells General Hospital. Resident House Surgeon; unmarried. Salary £100 per annum, with board, furnished apartments, gas, firing, and attendance. Applications to the Assistant Secretary by July 6th.

**Non-Resident Medical Officers.**

Bristol Dispensary, Castle Green, Bristol. Vacancy in the Medical Staff; doubly qualified. Applications to the Secretary by July 7th.

Chelsea Hospital for Women, Fulham Road, S.W. Pathologist-Physician to Out-patients. Applications, on forms to be obtained at the Hospital, to the Secretary by July 8th.

City of London Hospital for Diseases of the Chest, Victoria Park, E. Assistant Physician; must be Fellows or Members of the Royal College of Physicians of London. Applications to the Secretary, at the office, 24, Finsbury Circus, E.C., by July 5th.

Cookham Union. District Medical Officer. Salary £25 per annum. Applications to R. A. Ward, Clerk to the Guardians, Broadway, Maidenhead, by July 4th.

King's College Hospital. Anæsthetist. Applications to Mr. J. W. Cunningham, King's College, London.

Newburn District Local Board. Medical Officer of Health. Salary £30 per annum. Applications, marked "Application for Appointment of Medical Officer of Health," to G. Wilkinson, Clerk to the Board, 27, Mosley Street, Newcastle-upon-Tyne, by July 1st.

New Hospital for Women, 144, Euston Road, N.W. Two Clinical Assistants; must be fully qualified medical women. Appointments for one year. Applications to M. M. Bagster, Secretary, by June 30th.

Royal Victoria Hospital, Bournemouth. Ophthalmic Surgeon. Applications to the Secretary by July 1st; and a House-Surgeon-Secretary. Salary £100 per annum and board for the latter. Applications to the Chairman of Committee by July 1st.

St. Bartholomew's Hospital and College. Demonstrator of Chemistry and Assistant Demonstrator of Chemistry. Applications to Dr. T. W. Shore, Warden, by June 30th.

University College, London. Assistant Surgeon. Applications to the Secretary by July 4th.

